# Woody vegetation dynamics in the tropical and subtropical Andes from 2001 to 2014: Satellite image interpretation and expert validation

**DOI:** 10.1111/gcb.14618

**Published:** 2019-04-07

**Authors:** T. Mitchell Aide, H. Ricardo Grau, Jordan Graesser, Maria Jose Andrade‐Nuñez, Ezequiel Aráoz, Ana P. Barros, Marconi Campos‐Cerqueira, Eulogio Chacon‐Moreno, Francisco Cuesta, Raul Espinoza, Manuel Peralvo, Molly H. Polk, Ximena Rueda, Adriana Sanchez, Kenneth R. Young, Lucía Zarbá, Karl S. Zimmerer

**Affiliations:** ^1^ Department of Biology University of Puerto Rico San Juan Puerto Rico; ^2^ Instituto de Ecología Regional CONICET‐Universidad Nacional de Tucumán Tucumán Argentina; ^3^ The Department of Earth and Environment Boston University Boston Massachusetts; ^4^ Department of Environmental Sciences University of Puerto Rico San Juan, Puerto Rico; ^5^ Department of Civil and Environmental Engineering Duke University Durham North Carolina; ^6^ Sieve Analytics San Juan, Puerto Rico; ^7^ Instituto de Ciencias Ambientales y Ecológicas (ICAE) Universidad de Los Andes Mérida Venezuela; ^8^ Consortium for the Sustainable Development of the Andean Ecoregion (CONDESAN) Quito Ecuador; ^9^ Palaeoecology & Landscape Ecology, Institute for Biodiversity & Ecosystem Dynamics (IBED) University of Amsterdam Amsterdam Netherlands; ^10^ Centro de Competencias del Agua (CCA) Lima Peru; ^11^ Instituto Geofisicos del Peru (IGP) Lima Peru; ^12^ Department of Geography and the Environment University of Texas at Austin Austin Texas; ^13^ School of Management Universidad de los Andes Bogota Colombia; ^14^ Programa de Biología Universidad del Rosario Bogotá Colombia; ^15^ Departments of Geography and Rural Sociology, GeoSyntheSES Lab Pennsylvania State University State College Pennsylvania

**Keywords:** agriculture, coupled natural human systems, expert validation, forest loss and regeneration, MODIS satellite imagery

## Abstract

The interactions between climate and land‐use change are dictating the distribution of flora and fauna and reshuffling biotic community composition around the world. Tropical mountains are particularly sensitive because they often have a high human population density, a long history of agriculture, range‐restricted species, and high‐beta diversity due to a steep elevation gradient. Here we evaluated the change in distribution of woody vegetation in the tropical Andes of South America for the period 2001–2014. For the analyses we created annual land‐cover/land‐use maps using MODIS satellite data at 250 m pixel resolution, calculated the cover of woody vegetation (trees and shrubs) in 9,274 hexagons of 115.47 km^2^, and then determined if there was a statistically significant (*p* < 0.05) 14 year linear trend (positive—forest gain, negative—forest loss) within each hexagon. Of the 1,308 hexagons with significant trends, 36.6% (*n* = 479) lost forests and 63.4% (*n* = 829) gained forests. We estimated an overall net gain of ~500,000 ha in woody vegetation. Forest loss dominated the 1,000–1,499 m elevation zone and forest gain dominated above 1,500 m. The most important transitions were forest loss at lower elevations for pastures and croplands, forest gain in abandoned pastures and cropland in mid‐elevation areas, and shrub encroachment into highland grasslands. Expert validation confirmed the observed trends, but some areas of apparent forest gain were associated with new shade coffee, pine, or eucalypt plantations. In addition, after controlling for elevation and country, forest gain was associated with a decline in the rural population. Although we document an overall gain in forest cover, the recent reversal of forest gains in Colombia demonstrates that these coupled natural‐human systems are highly dynamic and there is an urgent need of a regional real‐time land‐use, biodiversity, and ecosystem services monitoring network.

## Graphical abstract

The interactions between climate and land‐use change are dictating the distribution of flora and fauna and reshuffling biotic community composition around the world. In the Andes of South America, we found a net increase in woody vegetation above 1000 m. While climate change has likely contributed to this increase, especially at higher elevations, land‐use change is the primary factor altering the contemporary distributions of many species.

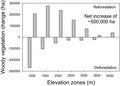

## 1 INTRODUCTION

Land‐cover change, particularly the distribution of woody vegetation, plays a key role in conservation of biodiversity and ecosystem services such as watershed and soil protection, carbon sequestration, and food production. Most research on land‐cover dynamics has focused on lowlands as they include the greatest proportion of land on earth, most of the human population, and the majority of agricultural production (Verburg et al., 2015). In contrast, there are fewer land change studies of mountain regions because topography and frequent cloud cover limit the use of remote sensing (Rudel, Sloan, Chazdon, & Grau, 2016). Nevertheless, mountain ecosystems harbor high biodiversity, endemism, agrobiodiversity, and cultural diversity, and they play key roles in regulating watershed and soil conservation in the sources of the most important rivers of the world (Viviroli, Dürr, Messerli, Meybeck, & Weingartner, 2007). A paramount example of this ecological relevance is the Andes of South America. The tropical and subtropical Andes extend for ~5,000 km and include many peaks >6,000 m, some of the world's most diverse biological communities, high cultural and agricultural diversity, and the most developed historical human societies in South America (Veblen, Young, & Orme, 2007).

The Andes mountains have been occupied for millennia, and historically, agropastoral activities have been the dominant influence on Andean ecological systems (Dantas, Figueroa, & Laguens, 2014; Etter, McAlpine, Wilson, Phinn, & Possingham, 2006; Hess, 1990). These activities have greatly reduced the area of forest cover in the past (Josse et al., 2011), and in many regions, deforestation continues (Armenteras, Rodríguez, Retana, & Morales, 2011; Fjeldså, Álvarez, Lazcano, & Leon, 2005; Hansen et al., 2013; Young, 1998). An important agropastoral activity in the Andes is subsistence agriculture. Andean subsistence agriculture is vulnerable to socioeconomic and climate changes because it is often practiced in marginal conditions and could consequently lead to abandonment and secondary forest recovery (Aide et al., 2013; Grau & Aide, 2008). Local and regional studies have described forest recovery in the Andes in Venezuela (Gutiérrez, Gärtner, López H., Pacheco, & Reif, 2013), Colombia (Sánchez‐Cuervo, Aide, Clark, & Etter, 2012), Bolivia (Redo, Aide, & Clark, 2012), and Argentina (Grau et al., 2008; Nanni & Grau, 2014), but there is still some skepticism about the generality of these dynamics (Farley, 2010).

Although studies in the Andes have associated forest recovery with decreasing rural population and a decline in agricultural activities (Aide & Grau, 2004; Grau & Aide, 2008), including grazing, others have argued that a decline in the rural population does not necessarily lead to forest recovery (Gray, 2009a; Radel, Schmook, & Chowdhury, 2010). Instead, the decline in local labor can be compensated by shifting from labor‐intensive agriculture to grazing, high input agriculture, mining, or the establishment of tree plantations, as well as agricultural mechanization in lower and mid‐elevation Andean sites (Zimmerer & Vanek, 2016). Furthermore, grazing could expand, reducing forest cover, if fire is used more frequently as a response to a decline in the availability of labor (Carilla & Grau, 2010). Forest cover could also decline if agriculture shifts to higher elevations due to increasing temperatures (Tito, Vasconcelos, & Feeley, 2018), or due to increasing demand for agricultural products as observed in the Southeast Asia Massif (Zeng, Gower, & Wood, 2018).

Along with the impacts of humans in the Andes, climate change and variability also have and will continue to play an important role in land‐cover and land‐use dynamics (Tovar, Arnillas, Cuesta, & Buytaert, 2013). Climate change has had diverse effects on ecosystems worldwide (Leemans & Eickhout, 2004; Moritz et al., 2008; Parmesan & Yohe, 2003; Pecl et al., 2017; Wiens, 2016). While the impacts in tropical regions have not been as dramatic as those described for the polar regions (Chapin et al., 2010; Massom et al., 2018; Paolo, Fricker, & Padman, 2015), researchers have documented shifts in the distributions of plants (Duque, Stevenson, & Feeley, 2015; Fadrique et al., 2018; Feeley et al., 2011; Morueta‐Holme et al., 2015), insects (Chen et al., 2009; Moret, Aráuz, Gobbi, & Barragán, 2016), birds (Campos‐Cerqueira, Arendt, Wunderle, & Aide, 2017; Forero‐Medina, Terborgh, Socolar, & Pimm, 2011; Freeman & Freeman, 2014), amphibians (Campos‐Cerqueira & Aide, 2017; Pounds et al., 2006; Pounds & Crump, 1994; Pounds, Fogden, & Campbell, 1999; Raxworthy et al., 2008; Seimon et al., 2017), dramatic declines in bird populations (Blake & Loiselle, 2015), changes in forest plant composition (Esquivel‐Muelbert et al., 2019), upslope shifts of crops including indigenous food plants (Zimmerer et al., 2018), and upward displacement of the forest–paramo ecotone (Rodríguez‐Morales, Chacón‐Moreno, & Ataroff, 2009). Furthermore, the flora and fauna of tropical mountains are especially susceptible to the effects of climate change because many species have limited altitudinal distributions and small changes in climate could result in local extinctions (Laurance et al., 2011).

Climate models (i.e., RCP4.5 and RCP8.5) predict increases in temperature up to 5°C by 2,100 in the central and southern Andes (Zazulie, Rusticucci, & Raga, 2017). This level of change will affect community composition (Ramirez‐Villegas et al., 2014) and the distributions and functioning of whole ecosystems (Dangles et al., 2017). For example, highland grasslands are warming (Tovar et al., 2013; Vuille, Bradley, Werner, & Keimig, 2003) and this is expected to lead to a dramatic decline in their extent (Buytaert, Cuesta‐Camacho, & Tobón, 2011). Andean wetlands are also changing in relation to climate‐induced glacier recession (Polk et al., 2017). These new abiotic conditions are expected to promote the encroachment of shrubs and trees into tropical montane grasslands and paramos (Helmer et al., 2019). In the Venezuelan Andes, these changes are predicted to decrease the area of paramo by 7 to 36% during the next 30 years (Suárez del Moral & Chacón‐Moreno, 2011). Furthermore, fire regimes are also expected to change in response to changes in climate and land‐use dynamics (Aráoz & Grau, 2010; Grau & Veblen, 2000; Holz et al., 2017; Uriarte et al., 2012).

Given the diversity of climates, habitats, and economic conditions across the Andes, we can expect a diversity of responses. For example, a decrease in forest cover is expected in regions where increasing temperatures force crops (e.g., coffee or potato) to higher elevations or where socioeconomic conditions promote rural development and new agricultural activity (e.g., Colombian Peace agreement). Better roads, stable socioeconomic conditions, and increase in the global demand for agricultural commodities could promote agricultural expansion in the foothills (e.g., oil palm, soybean, and sugar cane). In contrast, forest gains may occur if socioeconomic changes (e.g., urbanization) lead to rural out‐migration and/or the abandonment of pastures and agriculture followed by secondary succession. Alternatively, the expansion of plantations (e.g., cacao, coffee, eucalyptus) into abandoned pastures may be detected as forest expansion. At the highest elevations (e.g., tropical alpine grasslands, paramo, puna), increasing temperatures could facilitate the encroachment of trees and shrubs. These scenarios highlight the urgent need to understand how the spatiotemporal interactions between human and natural systems are changing the distribution of biodiversity, ecosystem services, and socioeconomic environment in the Andes.

Here we document how land‐use patterns are changing in the subtropical and tropical Andes of South America as a consequence of the interaction between natural and human systems. We focus on the change in woody vegetation (i.e., shrubs and trees) above 1,000 m between 2001 and 2014, based on a land‐use classification derived from MODIS satellite data at 250‐m pixel resolution. Specifically, (1) we determine how the distribution of woody vegetation is changing at the scale of the Andes, within each country, and along the elevation gradient; (2) we relate changes in woody vegetation with country, elevation, slope, nighttime lights, and population change; and (3) we document the drivers of change in “hotspots” of forest loss and gain based on local expert knowledge, literature, and sources of high resolution imagery (e.g., Google Earth).

## 2 METHODS

### 2.1 Study region

**Figure 1:**
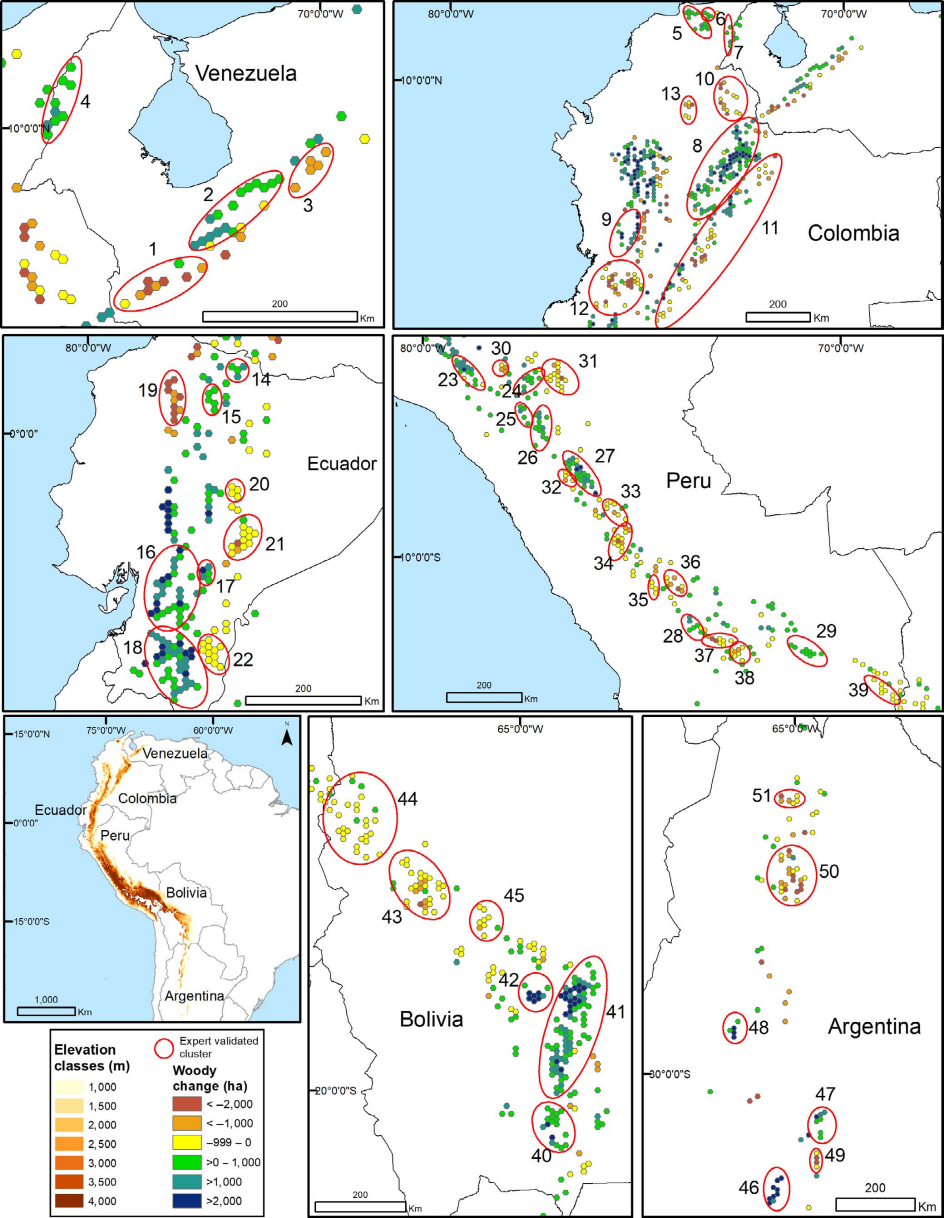
The distribution of elevation classes within the tropical and subtropical Andes and the hexagons that had a significant 14 year linear increase or decrease in woody vegetation in each country. Clusters of woody vegetation gain and loss (i.e., numbered circles) were evaluated by in‐country experts. The number associated with each cluster corresponds with information in Table 2]

The Andes of South America are the longest continental mountain range in the world, with many peaks above 6,000 m. The tropical and subtropical Andes, between 11° N and 33° S, spans approximately 5,000 km across six countries, and are one of the global regions of highest biodiversity as well as a major center of agro‐biodiversity. The study area extends from the Sierra Nevada de Santa Marta in northern Colombia to the province of San Luis, Argentina (Figure 1). The major biomes in this region are: tropical and subtropical moist broadleaf forest, tropical and subtropical dry broadleaf forest, montane grasslands and shrublands, and tropical and subtropical grasslands, savannas, and shrublands (Olson et al., 2001). The tropical Andes are the largest biodiversity hotspot in the world with >45,000 plant species and >3,000 vertebrate species (Myers, Mittermeier, Mittermeier, Fonseca, & Kent, 2000).

Human populations have played an important role in the transformation of Andean environments, with civilizations beginning more than 2,000 years ago (Burger, 1992) and achieving a large geographic influence, with Inca empire spreading from southern Colombia to Chile. Today the major cities of Merida in Venezuela, Medellin, and Bogota in Colombia, Quito and Cuenca in Ecuador, Arequipa, Ayacucho, and Cuzco in Peru, La Paz/El Alto, Cochabamba, Oruro, and Tarija in Bolivia, and Jujuy and Salta in Argentina all occur above 1,000 m in the Andes.

The Andes mountains have also been an important center for agriculture domestication (e.g., potatoes, quinoa, tomatoes, chili peppers, cotton, coca, tobacco, peanuts) (Piperno, 2011). Today, while a high diversity of crops continues to be cultivated in the Andes, maize and potatoes are by far the most important (Tito et al., 2018; Zimmerer et al., 2018).

### 2.2 Land‐use classification

The maps used in this study were a subset of annual land‐cover maps created for Latin America and the Caribbean for the period 2001 to 2014. Following methods outlined elsewhere (Aide et al., 2013; Clark, Aide, & Riner, 2012; Graesser, Aide, Grau, & Ramankutty, 2015; Nanni et al., 2019), Random Forest land‐cover classification models defined for each biome in Latin America and the Caribbean (Olson et al., 2001) were used to classify the following land‐cover categories in the MODIS imagery: cropland, pastureland, woody (including both natural tree cover and shrubs), plantations, and other (i.e., bare soil, ice, snow, rock, sand dunes, built‐up structures, and water).

Annual land‐cover maps were produced by classifying the MODIS satellite MOD13Q1 Vegetation Indices 250 m product for the period 2001–2014. The product is a 16 d composite of the highest quality pixels from daily images and includes the Enhanced Vegetation Index (EVI), blue (459–479 nm), red (620–670 nm), near infrared (NIR: 841–876 nm), and mid‐infrared (MIR: 2,105–2,155 nm) reflectance and pixel reliability, with 23 scenes per year. For each pixel, we calculated the mean, standard deviation, minimum, maximum and range for EVI, and blue, red, NIR, and MIR reflectance values from each year between 2001 and 2014. These statistics were calculated for all 12 months, two 6 month periods, and three 4 month periods. The pixel reliability layer was used to remove all unreliable samples (value = 3) prior to calculating statistics. These statistics were used as predictive variables in the Random Forest classifier.

Training data for each classifier were collected by overlaying a grid of MODIS pixels (250 m × 250 m) onto multitemporal high‐resolution imagery in Google Earth and registering the land‐cover class and date. More than 60,000 MODIS pixels were labeled to create the classification models used in this study. These data were associated with the pixel statistics to create a Random Forest classification model for each mapping zone. The mapping zone boundaries followed ecoregion and biome delineations. To train a zone‐specific Random Forest model, land‐cover samples within the mapping zone of interest and the samples’ Google Earth high‐resolution image acquisition date were paired with MODIS time series variables. For example, samples collected from 2005 Google Earth high‐resolution imagery were paired with 2005 MODIS time series variables. The zone‐specific Random Forest models were then applied annually to produce 14 annual land‐cover maps for each mapping region.

This study focused on the woody class (i.e., trees and shrubs) and the overall postclassification accuracy for the woody/non‐woody classification within the Andes was 94%. Accuracy was evaluated by comparing random pixels from the 2013 classification map with high‐resolution imagery from 2013 in Google Earth. For this study, a hexagon grid was placed over each annual land‐use classification map. Each hexagon had a size of 11 km (north to south) and an area of 115.47 km^2^ (~11,547 ha). All hexagons with a median elevation ≥1,000 m that intersected with the tropical and subtropical moist broadleaf forest, tropical, and subtropical dry broadleaf forest, montane grasslands and shrublands, and tropical and subtropical grasslands, savannas, and shrublands biomes in South America were included. A few hexagons occurred in Brazil and Venezuela, which were clearly not part of the Andes and they were eliminated. For each hexagon, we summed the area of all MODIS pixels classified as woody vegetation (trees or shrubs) for each year. The 14 years of woody vegetation area were used in a simple linear regression against time (i.e., year). Only hexagons with a statistically significant linear trend (*p* > 0.05, positive—forest gain, negative—forest loss) were included in the analyses. For these hexagons with a significant 14 year trend, we report the net change in woody vegetation between 2001 and 2014. This multiyear multipixel approach ensured that significant hexagons represented regions where there were long‐term (i.e., 14 year) directional changes in woody cover, rather than pixel‐level year to year fluctuation in a cover class due to droughts or fire. To capture the variability in land use along the elevation gradient, the patterns of woody vegetation gain and loss were summarized within seven elevation zones (1,000–1,499 m, 1,500–1,999 m, 2,000–2,499 m, 2,500–2,999 m, 3,000–3,499 m, 3,500–3,999 m, and >4,000 m).

### 2.3 Expert opinion

For each country, we visually identified clusters of hexagons with significant trends of forest loss and forest gain. For each cluster, in‐country experts (i.e., authors) determined if there was sufficient information to evaluate the cluster. Potential sources of information included: high‐resolution images in Google Earth, local or regional published studies, a global forest/no forest map based on Landsat 30 m resolution images (Hansen et al., 2013), and direct observations by the experts. If there was sufficient information to evaluate a cluster, the expert: (1) determined if the information supported or contradicted the MODIS classification; (2) determined the major driver of the observed changes; and (3) provided the source(s) of information used to evaluate the cluster of hexagons. In the Andes, pastures and natural grassland cover extensive areas and both cover types are actively grazed. In our classification these areas were included as a single class because it is difficult to distinguish them. In general, pastures mainly occurred in forested biomes (e.g., tropical moist forest) and an important driver of an increase in woody vegetation in these biomes was pasture abandonment. This was verified by reviewing images from previous years in Google Earth. In contrast, shrub invasion was a common cause of an increase in woody vegetation in natural grassland and this occurred predominantly at high elevation in the tropical montane grassland biome.

### 2.4 Environmental and socioeconomic variables

To determine the socioeconomic and environmental variables associated with deforestation and reforestation trends in the Andes we performed a logistic regression analysis in R using the glmulti package (Calcagno & Mazancourt, 2010). For this analysis, we used the 1,308 hexagons that had a statistically significant 14 year trend of forest loss (0) or gain (1) as the dependent variable and country, elevation class, mean slope, change in nighttime lights, and change in rural population as the independent variables. Mean slope of each hexagon was calculated with the slope spatial analysis tool in ArcGIS 10.6 using the SRTM 90m Digital Elevation Database v4.1 downloaded from http://srtm.csi.cgiar.org. The change in nighttime light (NTL) between 2001 and 2011 was taken from Andrade‐Núñez and Aide (2018) who analyzed NTL change for South America. We extracted the change in NTL for the Andes study region and aggregated the data to the hexagon level. The municipality level (i.e., third administrative unit) population change data set was created by Andrade‐Núñez and Aide (2018). Rural and urban population data were obtained from the last two census for each country from Redatam (http://www.redatam.org/redatam/en/index.html) and national census webpages and were extrapolated to 2001 and 2011. A detailed explanation of the methodology is described in Andrade‐Núñez and Aide (2018). The municipality population data was rescaled to the hexagon level.

## 3 RESULTS

**Figure 2:**
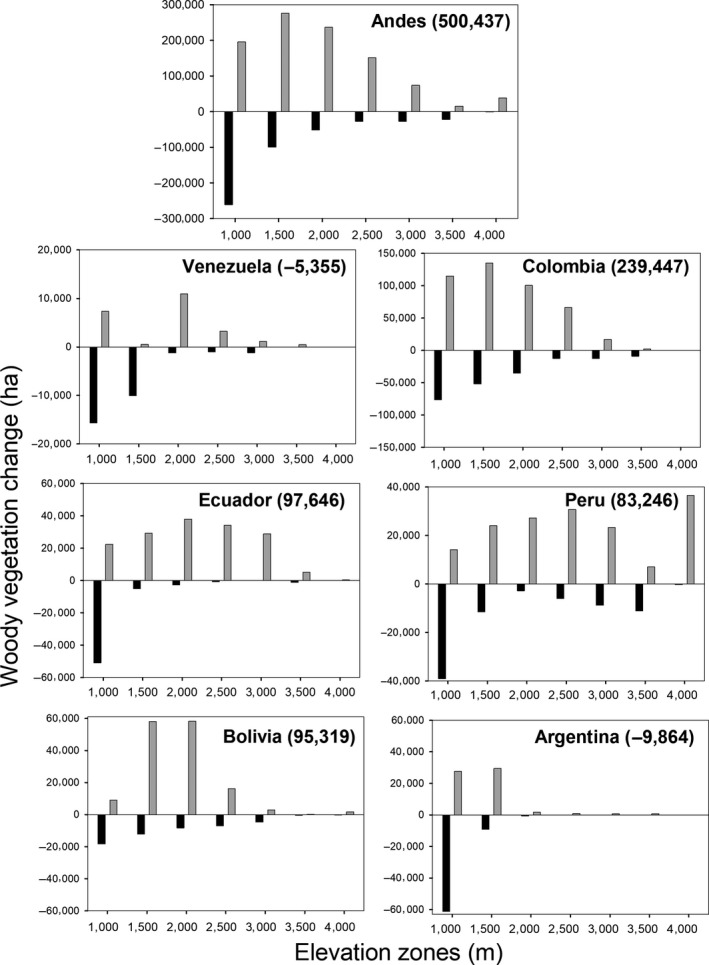
Gains and losses of woody vegetation from hexagons that had a significant linear 14 year negative or positive trend in the different elevation zones for the complete study region (Andes) and the six countries. The elevations zones were: 1,000–1,499 m, 1,500–1,999 m, 2,000–2,499 m, 2,500–2,999 m, 3,000–3,499 m, 3,500–3,999 m, and ≥4,000 m. The values in parenthesis are the net change for all elevation

The study region included 9,274 hexagons (~1,000,000 km^2^) and 1,308 had a significant trend; 36.6% (*n* = 479) lost forests and 63.4% (*n* = 829) gained forests. This resulted in a net gain of woody vegetation above 1,000 m in the Andes (Figure 2, Table 1). When we restricted the analyses to hexagons with significant linear trends over the 14 year study period, there was 488,353 ha of forest loss and 988,790 ha of forest gain (Table 1). The 1,000–1,499 m elevation zone had the greatest area of forest loss, while the 1,500–1,999 m and 2,000–2,499 m elevation zones had the greatest area of forest gain (Figure 2, Table 1). The amount of forest gain or loss was less than 2% of the total area within all elevation zones over the 14 year period (Table 1). It is notable that even above 4,000 m, in areas of native highland grasslands, there were hexagons with significant increases in woody vegetation.

**Table 1:** The absolute area and the % of the area of each elevation zone with significant woody vegetation loss or gain

Elevation zone (m)	Woody loss (ha)	% loss	Woody gain (ha)	% gain	Net change (ha)
1,000–1,499	−261,265	−1.0	195,679	0.7	−65,586
1,500–1,999	−99,630	−0.6	276,485	1.6	176,855
2,000–2,499	−51,020	−0.4	236,612	1.7	185,592
2,500–2,999	−26,964	−0.2	151,834	1.3	124,870
3,000–3,499	−27,158	−0.3	74,041	0.7	46,883
3,500–3,999	−21,697	−0.2	15,512	0.1	−6,185
>4,000	−619	0.0	38,627	0.3	38,008
Total	−488,353		988,790		500,437

There were important differences at the country scale. For example, Colombia, Ecuador, Peru, and Bolivia had net gains in woody vegetation above 1,000 m, while Argentina and Venezuela had net losses (Figure 2). In all countries, most notably in Argentina, Ecuador, and Peru, the majority of forest loss occurred in the 1,000–1,499 m elevation zone. In contrast, forest gain occurred across a wider range of elevation zones (Figure 2). These gains in woody vegetation were mainly due to woody vegetation replacing areas that were previously classified as herbaceous (i.e., pasture/grasslands) (Figure S1).

Forest gain (*n* = 25) and forest loss (*n* = 26) clusters were evaluated by in‐country experts (Figure 1). These clusters included a total of 849 hexagons with significant positive or negative woody vegetation trends during the 14 years of the study. Expert opinion agreed with the remote sensing analysis (agreed with 48 clusters (94%) and disagreed with 3 (6%)). The most common land‐cover category replacing forests were pastures and crops, while forest gain often occurred following the abandonment of pasture and crops associated with rural–urban migration, shrub invasion/expansion in the highlands, and establishment of plantations (e.g., pine/eucalyptus) and crop expansion (e.g., shade‐grown coffee) (Table 2).

**Table 2:** Drivers of forest cover change of the 51 hotspots of forest loss and forest gain confirmed by experts from each country

Country	Cluster #	Drivers of forest gain						Drivers of forest loss					
		Pasture and agricultural abandonment	Highland shrub invasion	Pine/eucalyptus plantations	Shade coffee	Agroforestry	Unknown	Pasture expansion	Agriculture expansion	Mixed (roads, mines, pastures, agriculture)	Fire	Unknown	Data source
Venezuela	1, 3							x					1, pers. obs.
Venezuela	2, 4		x										2, 3
Colombia	5, 7				x								4, pers. obs.
Colombia	6						x						5
Colombia	8	x											6
Colombia	9					x							5
Colombia	10, 11, 12							x					5, 7, 8
Colombia	13									x			1, 5
Ecuador	14						x						1, 9, 10
Ecuador	15, 16, 17			x									1, 9, 10, 11, 12
Ecuador	18	x											1, 9, 10, 13
Ecuador	19							x					1, 9, 10, 14, pers.obs.
Ecuador	20								x				1, 9, 10, 15
Ecuador	21, 22									x			1, 9, 10, 16
Peru	23, 25–29		x										1
Peru	24	x											1
Peru	30–32, 34–37							x					1, 17
Peru	33, 39									x			1
Peru	38											x	1
Boliva	40, 41, 42	x											1
Boliva	43, 44, 45								x				1
Argentina	46, 47, 48	x											1
Argentina	49										x		1, pers. obs.
Argentina	50, 51								x				1, 18, 19, pers. obs.
Total		9	8	3	2	1	2	13	6	5	1	1	

Hotspots included regions with multiple adjacent hexagons with similar trends of forest cover change, and in regions where experts have knowledge of land‐use dynamics. The location of each region is highlighted in Figure 1. The data sources include: (1) high‐resolution images in Google Earth, (2) Suárez del Moral and Chacón‐Moreno (2011), (3) Rodríguez‐Morales et al. (2009), (4) FNC (2017), (5) González et al. (2018), (6) León‐Escobar (2011), (7) Observatorio de Drogas de Colombia (2018), (8) DANE (2014), (9) MAE (2015), (10) MAE (2017), (11) Jokisch (2002), (12) Jokisch and Lair (2002), (13) Oñate‐Valdivieso and Sendra (2010), (14) Baquero and Peralvo ( 2016), (15) Van Der Hoek (2017), (16) Curatola Fernández et al. (2015), (17) Hansen et al. (2013), (18) Gasparri and Grau (2009), (19) Nanni and Grau (2014).

A logistic regression analysis to test if forest loss or gain was related to country, elevation class, slope, change in nighttime lights, or change in rural population showed that elevation class and country were the most important variables among all models (Table 3). Overall the proportion of forest loss to gain shifted from loss dominating in the 1,000–1,499 m class and gain dominating above 1,500 m, but this pattern varied among countries (Figure 2). After controlling for elevation class and country, the next most important variable was the change in rural population between 2001 and 2011, and forest gain was associated with a decline in the rural population.

**Table 3:** Summary of the best models with forest loss or gain as the dependent variable and country, elevation class, slope, change in nighttime lights, and change in rural population within each significant hexagon as the independent variable

Model	AIC	∆AIC	Model weight
Elevation class + country	1,649.53	0	0.244
Elevation class + country + ∆ rural population	1,649.58	0.05	0.237
Elevation class + country + ∆ rural population + ∆ NTL	1,651.10	1.57	0.111
Elevation class + country + ∆ NTL	1,651.11	1.58	0.110

## 4 DISCUSSION

Patterns of woody cover change in the Andes varied along the elevation gradient and among countries. The overall pattern of an increase in woody vegetation, particularly at higher elevations (>1,500 m), is consistent with the expected effects of rural–urban migration (Aide & Grau, 2004), climate change, specifically increasing temperature (Feeley et al., 2011; Song et al., 2018), and the abandonment of marginal (i.e., low productivity) pasturelands and croplands (Curtis, Slay, Harris, Tyukavina, & Hansen, 2018; Grau & Aide, 2007, 2008 ). Land‐use change in the Andes between 2001 and 2014 resulted in the loss of ~500,000 ha and a gain of ~1,000,000 ha of woody vegetation cover, emphasizing the importance of land‐cover redistribution as a process at least as important as the overall net change (Aide et al., 2013; Nanni & Grau, 2014). In the foothills of the Andes (1,000–1,500 m), the overall pattern was forest loss mainly caused by an increase in pastures and croplands. Above 1,500 m, the dominant pattern was forest gain mainly due to abandoned pastures and small‐scale agriculture associated with rural–urban migration and woody vegetation densification and encroachment into the montane grasslands and paramo. In addition, the expansion of shade coffee cultivation and pine and eucalyptus plantations were also responsible for increases in woody vegetation in some areas of Colombia and Ecuador. This net gain in woody vegetation may provide opportunities for biodiversity conservation and the recovery of environmental services such as watershed protection and carbon sequestration (Chazdon et al., 2016; Grau & Aide, 2008), but the details and spatial patterns of these dynamics are complex because they reflect the dynamic interplay between natural (e.g., climate, topography) and human (e.g., migration, agriculture markets) systems, and these interactions can vary greatly along the elevation gradient and within and among the six countries included in this study.

### 4.1 Variation along the elevation gradient

In the 1,000–1,499 m elevation class, all countries except Colombia had greater loss of woody vegetation than gain, and Colombia, Ecuador, and Argentina were the countries that lost the greatest area (Figure 2). Forests were replaced mainly by pastures for cattle grazing, but also for mechanized croplands (e.g., sugar cane, soybeans, fruit orchards in Argentina) (Gasparri & Grau, 2009; Nanni & Grau, 2014).

Above 1,500 m the dominant dynamic was an increase in woody vegetation due to the abandonment of pasture and agricultural lands, similar to patterns observed globally (Curtis et al., 2018). In most cases, this was associated with out‐migration; including the dramatic case of Colombia where violence displaced more than 7 million rural people (UN Refugee, 2018), but most commonly due to working age out‐migration looking for better jobs, education, and health care in national cities (e.g., medium and large cities of Colombia—Lozano‐Gracia, Piras, Ibáñez, & Hewings, 2010, Bolivia‐Redo et al., 2012); or in other countries, (particularly important in Ecuador, Jokisch & Lair, 2002). In general, these changes suggest a trend of land‐use disintensification and woody vegetation regrowth in many areas, with intensive farming in peri‐urban locales and selective hotspots of commercial agriculture (Zimmerer, Carney, & Vanek, 2015). Another important driver of woody vegetation increase above 1,500 m was the expansion of shade coffee cultivation and the development of silvopastoral and conservation projects (e.g., payment for ecosystem services) in Colombia (León‐Escobar, 2011) and pine or eucalyptus plantations in Ecuador (Farley, 2010). In the highest elevation zones, the increase in woody vegetation in all countries also points to increasing temperatures, and possibly drier conditions facilitating shrubs encroachment and tree invasions above treeline into highland grasslands (e.g., Peru) and paramos (e.g., Venezuela), and grasslands of Argentina (Grau &Veblen, 2000). These results are consistent with an observed gain in canopy tree and net bare ground loss in mountain regions worldwide (Song et al., 2018). Although these higher elevation habitats have some agriculture (e.g., potatoes, wheat, quinoa), and grazing, if out‐migration reduces these activities and the use of fire, this could create a positive feedback accelerating shrub encroachment (Aráoz & Grau, 2010; Lambin & Meyfroidt, 2010; Lutz, Powell, & Silman, 2013).

### 4.2 Variation among countries

In Venezuela, woody vegetation loss (Figure 1, clusters 1, 3) mainly occurred in the 1,000–1,500 m elevation zone and the major driver of loss was pastures replacing shade coffee. In contrast, woody vegetation gain (e.g., clusters 2, 4) occurred at higher elevations (2,000–4,500 m) and the most likely driver was shrub densification at the cloud forest/paramo ecotone (Rodríguez‐Morales et al., 2009; Suárez del Moral & Chacón‐Moreno, 2011). These areas occur within or near national parks where access is difficult.

Land‐use change in the Andes of Colombia was very dynamic. It was the country with the greatest net increase in woody vegetation, but it also had the greatest loss in woody vegetation. Forest loss in the 1,000–1,499 m elevation class was mainly due to pasture expansion (e.g., clusters 10–12), while most forest gain occurred above 1,500 m associated with rural–urban migration and abandonment of pastures and agricultural lands (e.g., cluster 8 north of Bogota, Rubiano, Clerici, Norden, & Etter, 2017). Increases in woody vegetation cover was also associated with a shift in the distribution of coffee cultivation in favor of regions that produce higher quality and eco‐friendly coffee (e.g., clusters 5, 7, Rueda & Lambin, 2013, FNC, 2017) and silvopastoral projects, that promoted the introduction of foraging tree species into cattle pastures (e.g., cluster 9, Calle, Murgueitio, & Chará, 2012). However, it is important to note that although we detected gains in woody vegetation over the 14 year study period, in some areas these forests are being transformed again into pasture and agriculture lands. The recent increase in deforestation in Colombia, mainly in the lowlands (<1,000 m), has been associated with changing dynamics related to the postconflict peace agreement (Clerici et al., 2018). Given that rural development incentives are an important component of the agreement, the trend of increasing forest cover is likely to be reversed as pastures and croplands expand in the Andes.

Ecuador clearly demonstrates the overall pattern documented for the Andes with woody vegetation loss in the foothills (1,000–1,499 m) and gain above 1,500 m (Figure 1). Hotspots of loss occurred in the north on the western flank of the Andes (cluster #19), where pastures expanded and along the eastern flank of the Andes (Figure 1, clusters 20–22) where new roads into the Amazon lowlands are facilitating mining activities and pasture and agriculture expansion. The large area of woody vegetation gain in the province of Loja in southern Ecuador (cluster 18, Gray, 2009b) was associated with out‐migration, to other countries and into the Amazon lowlands. Although the abandonment or reduction of human pressure on the environment is a critical component of forest gain, given that much of this region occurs in the tropical dry forest biome, increase precipitation associated with El Niño events in 1991/1992, 1994/1995, 1997/1998, and 2004/2005 (Bendix & Bendix, 2006) may have contributed to the increase in woody vegetation. New pine and eucalyptus plantations (clusters 15–17, Jokisch, 2002; Jokisch & Lair, 2002) explain forest expansion in other regions above 2,000 m in Ecuador.

In Peru, the regions of forest loss were generally below 2,000 m (Figure 1 clusters 30, 31, 33, 36, 37) where forest were replaced by pastures, often as a consequence of new roads providing access to Amazon lowlands in Amazonas, Cusco, Madre de Dios, and Puno (Glinskis & Gutiérrez‐Vélez, 2019; Potapov et al., 2015). Migration out of the highlands into the lowlands may link the forest gain and forest loss with long‐settled areas in the higher elevations being abandoned and new areas in the Amazonian frontiers being settled. Most woody vegetation gain was associated with areas above 2,000 (Clusters 23, 25–29) and the most common dynamic was shrub densification. In addition, in the Peruvian Cordillera Blanca, pioneer species have been documented colonizing area where glaciers have receded (Mark et al., 2017; Young, Ponette‐González, Polk, & Lipton, 2017).

In Bolivia, woody vegetation gain and loss showed a strong spatial segregation (Figure 1) with woody loss in the north and gain in the south. In the northern clusters 43–45 in the La Paz and Cochabamba Departments (the “media luna” region), government‐sponsored development and colonization policies have promoted migration from the highlands to the Andean foothills and this has resulted in an increase in small‐scale agriculture and coca production (Zimmerer, 2015). In the south (clusters 40–42), out‐migration within Bolivia (e.g., Santa Cruz) and internationally (e.g., Argentina) has led to pasture and small‐scale agriculture abandonment and secondary forest succession in the neighboring foothills.

Argentina was the country with the smallest total area in this study, yet it had the largest proportion of lowland deforestation, which was associated with the expansion of large‐scale agriculture (e.g., soybeans, sugarcane, blueberries, citrus) concentrated in the lowest hexagons where there is a rapid transition from the foothills to the plains that are more appropriate for mechanized agriculture (clusters 50–51) (Gasparri & Grau, 2009; Nanni & Grau, 2014). In contrast, regions of woody vegetation gain occurred at higher elevation (clusters 46–48), and the dominant dynamic appears to be the abandonment or a reduction in grazing and a shift in the local economy toward tourism.

### 4.3 Agricultural implications

Although the major transitions detected in our analyses were between areas classified as pastures and woody vegetation (i.e., shrubs or trees), the role of agriculture was not trivial. Expert opinion in the present study (Table 2) and a recent study of global forest loss drivers (Curtis et al., 2018) coincided in identified agriculture as an important driver of forest lost in the Andes. For example, expert opinion listed cropland expansion in ~ 25% of the clusters with woody vegetation loss (Table 2), mostly below 2,000 m and in association with crops for emerging markets (e.g., ethanol from sugar cane), niche crops (e.g., blueberries or coca), and subsistence agriculture along new roads into the lowlands of Ecuador, Peru, and Bolivia. In contrast, the decline in croplands was often associated with rural–urban migration. Rural out‐migration and the availability of nonagriculture jobs can discourage labor‐intensive agriculture, maintenance of terraces and irrigation systems, and time demanding herding leading to declining agricultural production. Furthermore, liberalization policies (e.g., free trade agreements) have reduced the costs of many imported foods (e.g., maize imported from the US), discouraging farmers from cultivating staples for the domestic market (Hazell, Poulton, Wiggins, & Dorward, 2010). Although the loss of croplands may contribute to new forests and increase habitat for many species above 1,500 m, it has negative impacts of the people that remain by reducing their agrodiversity (e.g., nutritional diversity and adaptive capacity of local food plants to climate change) (Zimmerer et al., 2018).

### 4.4 Biodiversity and conservation implications

The change in the distribution of forests in the Andes could have important repercussions for the biota of the world's largest biodiversity hotspot (Myers et al., 2000), especially where it entails the conversion of mature forests (Watson et al., 2018). Species which have had less success adapting to changing conditions have often been range‐restricted species with limited ecological plasticity (Sekercioglu, Schneider, Fay, & Loarie, 2008); a description that captures much of the diversity in the Andes. Understanding how species will respond to climate change is a fundamental step for effective biodiversity conservation, but land‐cover and land‐use change must be considered given that it is the primary factor altering the contemporary distributions of many species and restricting their adaptative response to climate change by migration.

### 4.5 Climate–vegetation interactions

One possible implication of climate warming at high elevations is the reduction of cloudiness (Barros, 2013), which could have a strong impact on the vulnerability of forest ecosystems, especially at the highest elevations, independently of human activities. However, deforestation below 1,500 m also poses a significant threat to the climate–vegetation interactions. For example, evapotranspiration from Andean lowland forests is critical to establish strong daytime upslope moisture convergence that is necessary to form clouds and produce precipitation, but latent heat fluxes in the lower troposphere can impact convective activity and precipitation (Sun & Barros, 2015a,2015b). This suggests that continued lowland deforestation and warming could lead to drought amplification at higher elevations, which could increase fire frequencies and possibly limit treeline expansion (Harsch, Hulme, McGlone, & Duncan, 2009; Rehm & Feeley, 2015).

### 4.6 Data gaps and future directions

While remote sensing and global models will assist in predicting how forest will respond to a changing climate, it is much more complicated to predict how land‐use decisions (e.g., the pathways of agricultural expansion vs. disintensification) and fauna will respond (Pontius & Spencer, 2005). An important example is the forests in Colombia. While this study documented and increase in woody vegetation in the Andes of Colombia, recent reports have shown a rapid increase in deforestation in the lowlands (Hettler, Thieme, & Finer, 2017) associated with the Peace Agreement and the difficulties the government has faced in establishing a presence in remote regions. These dynamics are likely to reverse the forest gain process in the Andes documented in this study.

To monitor and respond to these widespread and rapid changes in the Andes there is an urgent need for a regional land‐use, biodiversity, and ecosystem services monitoring network. This will be a challenge given that fine‐grained/high spatial resolution land‐use maps do not exist at the scale of the Andes, demographic and socioeconomic data are collected sporadically, climate stations are scarce, and we do not have reliable distribution maps for the flora and fauna. Hopefully, we can overcome these challenges and do a better of managing and conserving the largest biodiversity hotspot in the world.
