# Supplementary material for: Woody vegetation dynamics in the tropical and subtropical Andes from 2001 to 2014: Satellite image interpretation and expert validation
Source: Glob Chang Biol. 2019 Apr 7;25(6):2112–26. doi: 10.1111/gcb.14618 (PMC6849738; doi:10.1111/gcb.14618)

Supplementary information.

Supplementary Figure S1. The relationship between the area of woody vegetation and grassland vegetation change in the hexagons that had a significant 14-year trend of deforestation or reforestation.


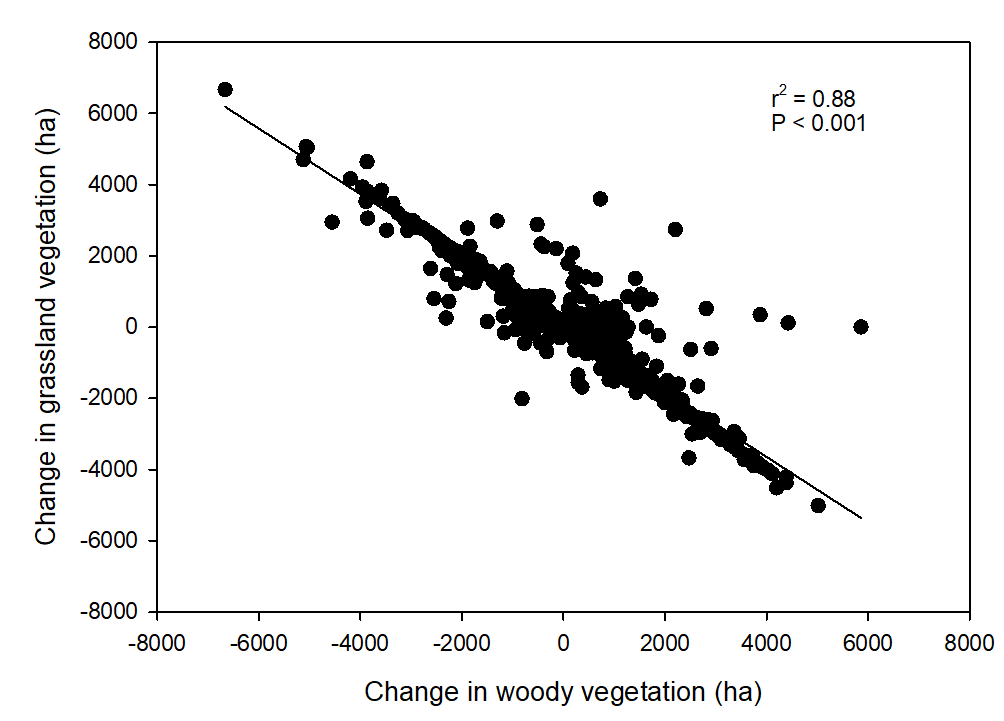

Supplement: Supplementary file 1 [file GCB-25-2112-s001.docx]
